# Different Effects of Three Selected *Lactobacillus* Strains in Dextran Sulfate Sodium-Induced Colitis in BALB/c Mice

**DOI:** 10.1371/journal.pone.0148241

**Published:** 2016-02-03

**Authors:** Yi Cui, Hongyun Wei, Fanggen Lu, Xiaowei Liu, Deliang Liu, Li Gu, Chunhui Ouyang

**Affiliations:** Department of Gastroenterology, The Second Xiangya Hospital of Central South University, Changsha, Hunan Province, China; CWRU/UH Digestive Health Institute, UNITED STATES

## Abstract

**Aim:**

To analyze the changes of different *Lactobacillus* species in ulcerative colitis patients and to further assess the therapeutic effects of selected *Lactobacillus* strains on dextran sulfate sodium (DSS)-induced experimental colitis in BALB/c mice.

**Methods:**

Forty-five active ulcerative colitis (UC) patients and 45 population-based healthy controls were enrolled. Polymerase chain reaction (PCR) amplification and real-time PCR were performed for qualitative and quantitative analyses, respectively, of the *Lactobacillus* species in UC patients. Three *Lactobacillus* strains from three species were selected to assess the therapeutic effects on experimental colitis. Sixty 8-week-old BALB/c mice were divided into six groups. The five groups that had received DSS were administered normal saline, mesalazine, *L*. *fermentum* CCTCC M206110 strain, *L*. *crispatus* CCTCC M206119 strain, or *L*. *plantarum* NCIMB8826 strain. We assessed the severity of colitis based on disease activity index (DAI), body weight loss, colon length, and histologic damage.

**Results:**

The detection rate of four of the 11 *Lactobacillus* species decreased significantly (*P* < 0.05), and the detection rate of two of the 11 *Lactobacillus* species increased significantly (*P* < 0.05) in UC patients. Relative quantitative analysis revealed that eight *Lactobacillus* species declined significantly in UC patients (*P* < 0.05), while three *Lactobacillus* species increased significantly (*P* < 0.05). The CCTCC M206110 treatment group had less weight loss and colon length shortening, lower DAI scores, and lower histologic scores (*P* < 0.05), while the CCTCC M206119 treatment group had greater weight loss and colon length shortening, higher histologic scores, and more severe inflammatory infiltration (*P* < 0.05). NCIMB8826 improved weight loss and colon length shortening (*P* < 0.05) with no significant influence on DAI and histologic damage in the colitis model.

**Conclusions:**

Administration of an *L*. *crispatus* CCTCC M206119 supplement aggravated DSS-induced colitis. *L*. *fermentum* CCTCC M206110 proved to be effective at attenuating DSS-induced colitis. The potential probiotic effect of *L*. *plantarum* NCIMB8826 on UC has yet to be assessed.

## Introduction

Ulcerative colitis (UC), a primary type of inflammatory bowel disease (IBD), is a chronic bowel disorder characterized by recurrent uncontrolled inflammation of the intestinal mucosa [1]. UC was perceived as a Western disease in the past, based on the high incidence and prevalence reported in industrialized countries (especially northern Europe and North America). However, the incidence and prevalence of UC has continued to increase in various regions around the world in recent decades; numerous developing countries traditionally considered as areas of low incidence are undergoing a dramatic rise in disease incidence [1,2]. Not surprisingly, UC is currently emerging as a global disease.

Increasing prevalence may partly account for the heavy health burden UC brings about; another crucial contributor could be patient requirements for lifetime care due to a lack of a guaranteed curative therapeutic regimen [3]. Conventional medical treatment of UC involves the administration of high-dose steroids and immunomodulators. Recently, significant advances have provided substantial insight into the etiology and pathogenesis of UC. The role of intestinal microbes in particular was shown to be of greater significance than previously considered [4]. Thus, novel therapies geared towards modulating the intestinal flora has become a research focus, a representative of which was the extensive use of probiotics in IBD, including UC [5].

The beneficial effects of probiotics in the induction of remission and the maintenance of UC have been examined in numerous animal experiments as well as in clinical trials [6–9]. As one of the most extensively commercially exploited probiotic bacteria, the effectiveness and safety of several *Lactobacillus* strains for supplementation have been evaluated in several studies [10–12]. However, to date, the understanding of these strains is still far from being sufficient for appropriate application in UC patients. The possible therapeutic efficacy of certain *Lactobacillus* strains in UC has yet to be assessed or identified further.

Thus, to identify more putative *Lactobacillus* species that may be of therapeutic value for UC, we undertook a study to investigate the changes of different *Lactobacillus* species in UC patients. Because species-specific primers can only be developed if there are known gene sequences containing highly variable regions of the universal *Lactobacillus* genes, we merely analyzed the changes in 11 common *Lactobacillus* species in UC patients compared with healthy controls using published *Lactobacillus* species-specific gene sequences. Based on this analysis, three *Lactobacillus* strains from three species were selected to assess the therapeutic effects on DSS-induced experimental colitis in BALB/c mice.

## Materials and Methods

### Participants

Eligible patients were recruited from the Gastroenterology Department of The Second Xiangya Hospital between April 2008 and May 2012. Forty-five active UC patients (26 males and 19 females; average age, 34 years) based on widely accepted diagnostic criteria, and 45 population-based healthy controls (30 males and 15 females; average age, 28 years) were enrolled in the study.

Participants were excluded if they had concurrent bacillary dysentery, ischemic enterocolitis, enterophthisis, or the use of antibiotics within 4 weeks prior to the sampling.

All human fecal samples were collected with written informed consent from patients prior to participation in the study. The protocols for the collection and analysis of the samples were approved by the Ethical Committee of the Second Xiangya Hospital of Central South University, in accordance with the current revision of the Helsinki Declaration.

### Collection and storage of fecal samples

Fresh fecal samples were collected from 45 patients and 45 healthy controls. All samples were placed in sterile, anaerobic ice boxes and immediately stored at –80°C until processing within 2 h.

### DNA extraction from fecal samples

DNA extraction was performed following the manufacturer’s instructions (QIAamp DNA Stool Mini Kit; Qiagen, Hilden, Germany). Ninety samples were processed by a single individual. The concentration of the DNA extracts was assessed by measuring the optical density at wavelengths of 230 nm, 260 nm, and 280 nm after dilution with deionized water. The purity of total DNA extracted was checked by electrophoresis on a 1% agarose gel.

### PCR screening of *Lactobacillus* species in which the detection rate significantly differed between UC patients and healthy controls

The primers used in the investigation are listed in Table 1. BLAST searches were performed to determine the specificity of the primers. For DNA extracts from each fecal sample, PCR amplification reactions (in a total volume of 20 μL) were performed on a PCR thermal cycler (PC-808; Astec, Fukuoka, Japan). Each reaction was performed in a 20 μL reaction volume containing 2 μL of template DNA, 2 μL of 25 mM MgCl_2_, 0.5 μL of 10 mM dNTPs, 5 μL of 10×PCR buffer, 10 pmol of each primer, and 1.0 U of Taq DNA polymerase (Promega, Madison, WI, USA). PCR was performed using the following parameters: an initial denaturation at 95°C for 3 min, followed by 36 cycles of 94°C for 30 s; 56°C for 40 s; 72°C for 40 s; and a final elongation period at 72°C for 5 min. The amplifications were confirmed by standard electrophoresis of the PCR products (5 μL) using 2% agarose gels (Sigma–Aldrich, St. Louis, MO, USA) prepared in 0.5× TBE buffer (45 mM Tris base, 45 mM boric acid, and 1 mM EDTA [pH 8.0]) and visualized by silver staining. A *Lactobacillus* species was considered to be detectable in the fecal samples of participants when primed PCR products were obtained. Thus, we screened out the *Lactobacillus* species that had significantly different detection rates between the patients and controls.

**Table 1:** Sequencing primers for the Lactobacillus species used in this study.

Species	Primer	Sequence (5ʹ-3ʹ)	Annealing temperature (°C)	Amplicon size (bp)
*L*. *crispatus*	LcrisF	AGCGAGCGGAACTAACAGATTTAC	58	154
	LcrisR	AGCTGATCATGCGATCTGCTT		
*L*. *gallinarum*	LgallF	CGGTAATGACGCTGGGGAC	56	128
	LcrisR	AGCTGATCATGCGATCTGCTT		
*L*. *fermentum*	LfermF	GCACCTGATTGATTTTGGTCG	58	317
	LactoR	GTCCATTGTGGAAGATTCCC		
*L*. *johnsonii*	LactoF	TGGAAACAGRTGCTAATACCG	56	322
	LjohnR	CAGTTACTACCTCTATCTTTCTTCACTAC		
*L*. *salivarius*	LsaliF	CGAAACTTTCTTACACCGAATGC	58	332
	LactoR	GTCCATTGTGGAAGATTCCC		
*L*. *acidophilus*	F_acid	GAAAGAGCCCAAACCAAGTGATT	56	85
	R_acid	CTTCCCAGATAATTCAACTATCGCTTA		
*L*. *casei*	F_case	CTATAAGTAAGCTTTGATCCGGAGATTT	56	132
	R_case	CTTCCTGCGGGTACTGAGATGT		
*L*. *delbrueckii*	F_delb	CACTTGTACGTTGAAAACTGAATATCTTAA	56	94
	R_delb	CGAACTCTCTCGGTCGCTTT		
*L*. *paracasei*	F_paca	ACATCAGTGTATTGCTTGTCAGTGAATAC	56	88
	R_paca	CCTGCGGGTACTGAGATGTTTC		
*L*. *plantarum*	F_plan	TGGATCACCTCCTTTCTAAGGAAT	56	80
	R_plan	TGTTCTCGGTTTCATTATGAAAAAATA		
*L*. *reuteri*	F_reut	ACCGAGAACACCGCGTTATTT	56	93
	R_reut	CATAACTTAACCTAAACAATCAAAGATTGTCT		
*E*. *faecalis*	Efs130F	AACCTACCCATCAGAGGG	56	360
	Efs490R	GACGTTCAGTTACTAACG		
*Lactobacillus* spp	F_alllact	TGGATGCCTTGGCACTAGGA	58	92
	R_alllact	AAATCTCCGGATCAAAGCTTACTTAT		
All bacteria	F_eub	TCCTACGGGAGGCAGCAGT	58	466
	R_eub	GGACTACCAGGGTATCTAATCCTGTT		

### Quantitative real-time PCR (qPCR) analysis of the *Lactobacillus* species in UC patients and healthy controls

The qPCR reaction was performed using an Applied Biosystems 7300 Real-Time system (Applied Biosystems, Foster City, CA, USA). Quantification assays and data analyses were performed using SDS version 1.2.3 software (Applied Biosystems). A 50 μL PCR reaction contained 5.0 μL of template DNA (50 ng), a 2.5 μL mixture of screened primers (10 pmol/L for each), 25.0 μL of 2× GoTaq qPCR Master Mix, 0.5 μL of 100× CXR reference dye, and sterile ddH_2_O. The qPCR reaction conditions were as follows: DNA polymerase activation at 95°C for 5 min, followed by 40 cycles of DNA melting at 95°C for 15 s and annealing at 56°C for 60 s. The specificity of the qPCR reaction was confirmed by melting-curve analysis.

The data were analyzed using the 2^-ΔΔCt^ method. As reported in several studies [13,14], we used total bacteria as the endogenous control to normalize the data. The threshold cycle (Ct) indicates the fractional number at which the amount of amplified target reaches a fixed threshold. ΔCt was calculated as the difference between the Ct value of the primers specific for *Lactobacillus* species and the Ct value of the primers for total bacteria. ΔΔCt is defined as the difference between the ΔCt value of UC patients and the ΔCt value of the control group. The fold change of expression of each Lactobacillus species in UC patients relative to that of the healthy controls was calculated using log10 RQ where RQ is 2^-ΔΔCt^. Log_10_RQ correlates directly to up-regulation (positive value) and down-regulation (negative value). A value of 1 in log_10_RQ (relative quantification) represents a 10-fold increase in expression. Similarly, a value of -1 represents 10-fold decrease in expression compared to control.

### Animal trial protocol

Sixty female (8-week-old) BALB/c mice weighing 20.0 ± 2.0 g were purchased from Hunan SJA Laboratory Animal Co. Ltd., Changsha, China. Each group consisted of ten mice. All mice were housed at a room temperature of 20–22°C, 50 ± 10% humidity, and a 12 h diurnal light/dark cycle. Throughout the trial period, the mice were fed standard rat chow and water *ad libitum*. All animal experimental procedures were reviewed and approved by the Institutional Animal Care and Use Committee of Central South University.

Three accessible standard *Lactobacillus* strains that were previously isolated and identified by our research institutions (preserved in the China Centre for Type Culture Collection) were selected as candidate *Lactobacillus* strains for further experimental effect verification. The candidate *Lactobacillus* strains were *L*. *fermentum* CCTCC M206110, *L*. *plantarum* NCIMB8826, and *L*. *crispatus* CCTCC M206119. The animals were randomly divided into three experimental groups (DSS-treated mice given 0.4 mL of 3.0×10^8^ colony forming units/mL of *L*. *fermentum* CCTCC M206110, *L*. *crispatus* CCTCC M206119, or *L*. *plantarum* NCIMB8826), three control groups (a healthy control group, a negative [normal saline] control group, and a positive [mesalazine; 6 mg/20 g] control group). Candidate strains of three *Lactobacillus* species, saline, or mesalazine were administered once daily via gavage from days 1–9. Between days 3 and 9, experimental colitis was induced via the consumption of 5% dextran sulfate sodium (DSS; Mw 40 kDa, MP Biomedicals Inc., Solon, OH, USA) in the drinking water [15].

Food, water/DSS consumption, body weight, fecal and urine output, and survival condition were monitored daily. The disease activity index (DAI) was determined by scoring the change in body weight, occult blood, and gross bleeding, as described previously [16]. On day 9, all mice were sacrificed by cervical dislocation. Then, the colons were removed and the colon length was measured. The distal colon was then collected and fixed in 10% buffered formalin for hematoxylin and eosin staining. Other parts of the colon were preserved in liquid nitrogen.

### Histologic analysis

Nine randomly selected fields (magnification 200×) were inspected in each section by two pathologists blinded to the treatment protocol. Grading of intestinal inflammation was determined as follows (Table 2). For each category of the score (inflammation, depth of lesions, and destruction of crypt), the points were multiplied by a factor of involvement of the visible epithelium (Table 2). The sum of the two category scores (inflammation and depth of lesions) were added to the inflammatory damage score (range, 0–24). The score of the destruction of the crypt category represents the crypt damage score (range, 0–16) [17,18].

**Table 2:** Histological score to quantify the degree of colitis.

Score	Inflammation	Depth of lesions	Destruction of crypt	Width of lesions (%)
0	None	None	None	
1	Mild	Submucosa	1/3 basal crypt	1–25
2	Severe	Muscularis	2/3 basal crypt	26–50
3		Sera	Intact epithelium only	51–75
4			Total crypt and epithelium	76–100

### Statistical analysis

All data are presented as the mean ± standard deviation (SD). Statistical package for social sciences (SPSS) software (version 17.0; SPSS Inc., Chicago, IL, USA) was used for data management and statistical analysis.

The detection rate of the different *Lactobacillus* species in UC patients and healthy controls was compared using a chi-square test. Weight, colon length, DAI scores, and histologic scores of all of the mouse groups were analyzed using an ANOVA test. Post hoc t-tests were conducted when the ANOVA values were significant. A *P*-value < 0.05 was considered to be statistically significant.

## Results

### The detection rate of 11 *Lactobacillus* species in UC patients and healthy controls

Using DNA extracts from fecal samples of 45 patients and 45 healthy controls, PCR amplification reactions were performed. The detection rates of the 11 *Lactobacillus* species in 45 patients and 45 healthy controls are listed in Table 3. Eight of the 11 *Lactobacillus* species were detectable in >50% of the healthy controls, and 5 of the 11 *Lactobacillus* species were detectable in >90% of the healthy controls. Two of the 11 *Lactobacillus* species (*L*. *johnsonii* and *L*. *casei*) were detectable in all of the healthy controls. Compared with the healthy controls, the detection rate of 4 of the 11 *Lactobacillus* species decreased significantly in the UC patients (*P* < 0.05). The four *Lactobacillus* species were *L*. *crispatus* (86.7% vs. 53.3%, *P* = 0.004), *L*. *fermentum* (95.6% vs. 35.6%, *P* = 0.001), *L*. *gasseri* (100.0% vs. 37.8%, *P* = 0.002), and *L*. *salivarius* (75.6% vs. 26.7%, *P* = 0.041). The detection rate of 2 of the 11 *Lactobacillus* species increased significantly in UC patients (*P* < 0.05). The two *Lactobacillus* species were *L*. *delbrueckii* (40.0% vs. 88.9%, *P* = 0.005) and *L*. *paracasei* (20.0% vs. 64.4%, *P* = 0.028).

**Table 3:** The detection rate of 11 Lactobacillus species in UC patients and healthy controls.

	UC patients (n = 45)		Healthy (n = 45)		p
	Numbers	%	Numbers	%	
*L*. *crispatus*	24	53.3	39	86.7	0.004
*L*. *gallinarum*	37	82.2	42	93.3	0.132
*L*. *fermentum*	16	35.6	43	95.6	0.001
*L*. *gasseri*	17	37.8	45	100	0.002
*L*. *salivarius*	12	26.7	34	75.6	0.041
*L*. *acidophilus*	38	84.4	42	93.3	0.206
*L*. *casei*	45	100.0	45	100.0	1.000
*L*. *delbrueckii*	40	88.9	18	40.0	0.005
*L*. *paracasei*	29	64.4	9	20.0	0.028
*L*. *plantarum*	19	42.2	26	57.8	0.090
*L*. *reuteri*	11	24.4	6	13.3	0.225
*Lactobacillus* spp	45	100	45	100	1.000

### Quantitative analysis of the *Lactobacillus* species in UC patients and healthy controls

**Fig 1:**
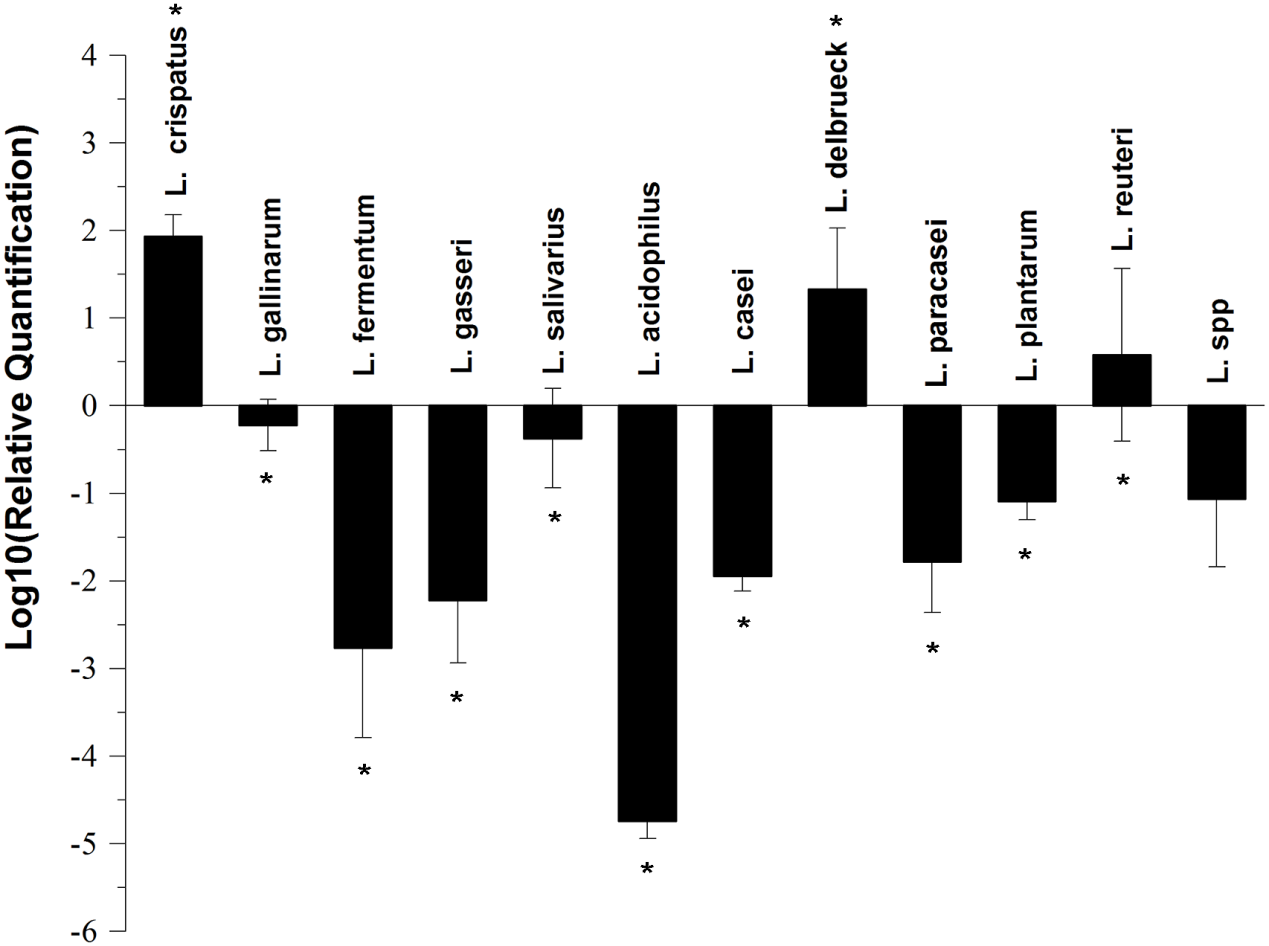
The relative quantification of *Lactobacillus* species in UC patients. The fold change was calculated using log_10_ RQ where RQ is 2^−ΔΔCt^. Statistical significance was determined by a *t*-test comparing ΔCt of healthy controls to ΔCt of UC patients. *P < 0.05.

The results of quantitative analysis of the 11 *Lactobacillus* species in UC patients and healthy controls are shown in Fig 1. Compared with the healthy controls, the number of *L*. *gallinarum*, *L*. *fermentum*, *L*. *gasseri*, *L*. *salivarius*, *L*. *acidophilus*, *L*. *casei*, *L*. *paracasei*, and *L*. *plantarum* decreased significantly in UC patients (*P* < 0.05), while the number of *L*. *crispatus*, *L*. *delbrueckii*, and *L*. *reuteri* increased significantly (*P* < 0.05).

### Effect of different *Lactobacillus* strains on weight loss in DSS-induced colitis in BALB/c mice

**Fig 2:**
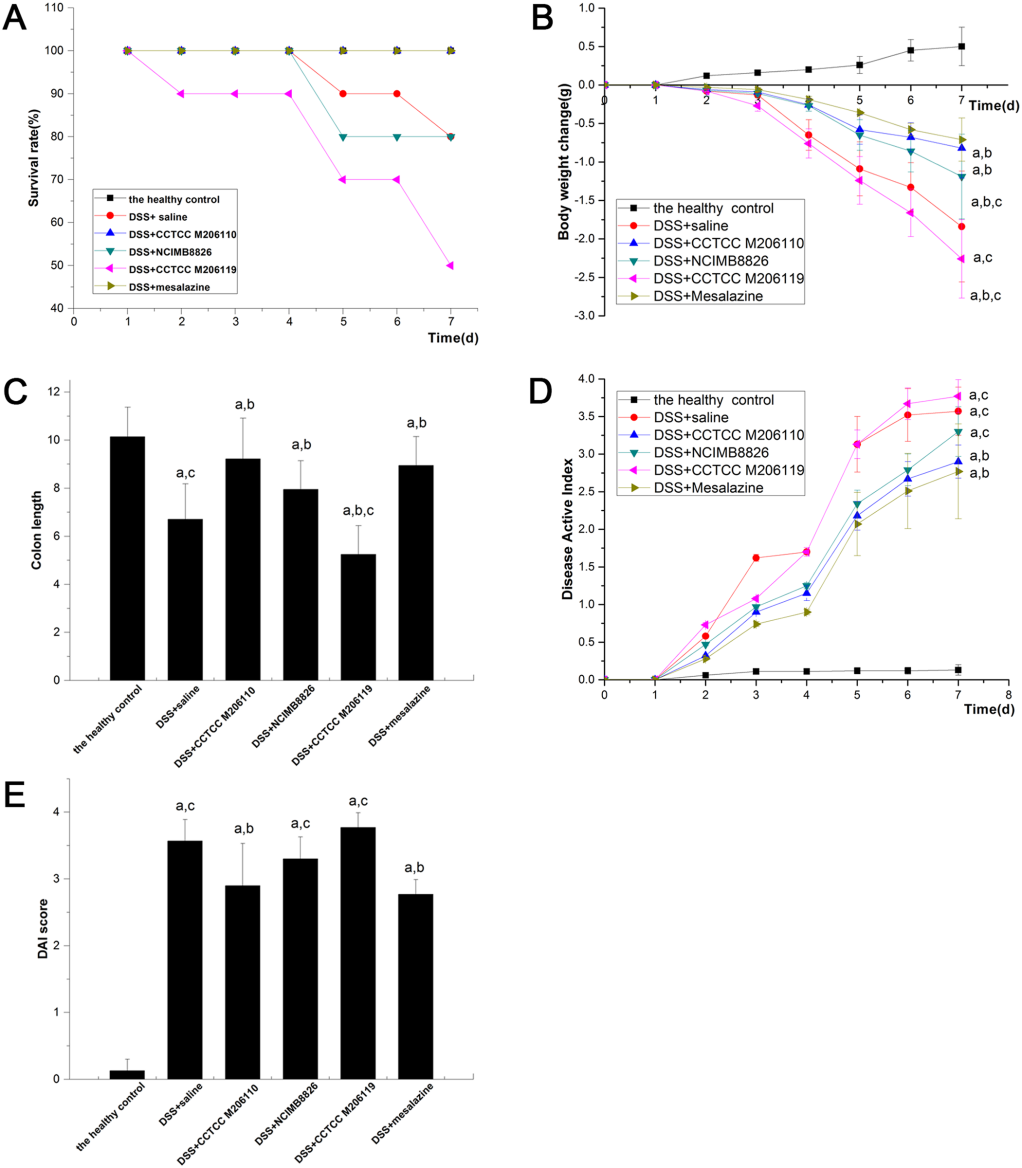
Effects of *Lactobacillus* strains NCIMB8826, CCTCC M206119, and CCTCC M206110 in dextran sulfate sodium-colitis mice. (A) Survival rate of six BALB/c mouse groups (*n* = 10) by the end of the experiment. (B) Effects of *Lactobacillus* strains treatments on colitis-induced body weight loss. The x-axis shows the mean weight (mean ± SD) recorded for each group on each day of the 7-day colitis induction. (C) Effects of *Lactobacillus* strain treatments on colitis-induced changes in colonic length. (D) Effects of *Lactobacillus* strains on colitis-induced changes in DAI scores. The daily mean DAI score ± SD is plotted for each group; E: Mean ± SD DAI scores on day 9. ^a^*P* < 0.05 vs. the healthy control group; ^b^*P* < 0.05 vs. the negative (saline) control group; ^c^*P* < 0.05 vs. the positive (mesalazine) control group.

Four days after inducing colitis, deaths began to occur due to gastrointestinal bleeding. The survival statuses of the animals at the end of the experiments are shown in Fig 2A. All groups exhibited significant weight loss during DSS treatment compared to the healthy control group, which was given water and a standard diet (*P <* 0.05), especially the negative control group. The positive control group had significantly less weight loss compared with the negative control group (-0.71 ± 0.28 g vs. -1.84 ± 0.72 g, *P* < 0.05). The weight loss in the *L*. *fermentum* CCTCC M206110 group (-0.82 ± 0.39 g vs. -1.84 ± 0.72 g, *P* < 0.05) and the *L*. *plantarum* NCIMB8826 group (-1.19 ± 0.55 g vs. -1.84 ± 0.72 g, *P* < 0.05) was also significantly less than the negative control group; however, the weight loss was significantly higher in the *L*. *crispatus* CCTCC M206119 group than in the negative control group (-2.26 ± 0.51 g vs. -1.84 ± 0.72 g, *P* < 0.05; Fig 2B).

### Effect of different *Lactobacillus* strains on colon length in DSS-induced colitis in BALB/c mice

All groups exhibited significant colon length reduction during DSS treatment compared to the healthy control group, which was given water and a standard diet (*P <* 0.05). The mesalazine group had significantly improved colon length shortening compared with the negative control group (9.18 ± 1.39 cm vs. 6.71 ± 1.47 cm, *P* < 0.05). In addition, the *L*. *fermentum* (9.22 ± 1.69 cm vs. 6.71 ± 1.47 cm, *P* < 0.05) and *L*. *plantarum* groups (7.95 ± 1.19 cm vs. 6.71 ± 1.47 cm, *P* < 0.05) both had significantly reduced shortening of the colon length compared with the negative control group. Furthermore, the *L*. *fermentum* group had more improvement in colon length shortening than the *L*. *plantarum* group (9.22 ± 1.69 cm vs. 7.95 ± 1.19 cm, *P* < 0.05); however, the results indicated that *L*. *crispatus* exacerbated the colon length shortening in comparison with the negative control group (5.25 ± 1.19 cm vs. 6.71 ± 1.47 cm, *P* < 0.05; Fig 2C).

### Effect of different *Lactobacillus* strains on DAI in DSS-induced colitis in BALB/c mice

All groups exhibited a significantly higher DAI score during DSS treatment compared to the healthy control group, which was given water and a standard diet (*P <* 0.05; Fig 2D). Compared with the negative control group, mesalazine treatment resulted in a significant decrease in the DAI score (2.77 ± 0.63 vs. 3.57 ± 0.32, *P* < 0.05). Moreover, the DAI scores of animals in the *L*. *fermentum* group were also significantly less than the negative control group (2.90 ± 0.22 vs. 3.57 ± 0.32, *P* < 0.05); however, the results indicated no significant difference in the DAI in the *L*. *plantarum* group or *L*. *crispatus* group compared with the negative control group (*P* > 0.05; Fig 2E).

### Effect of different *Lactobacillus* strains on histologic changes in DSS-induced colitis in BALB/c mice

**Fig 3:**
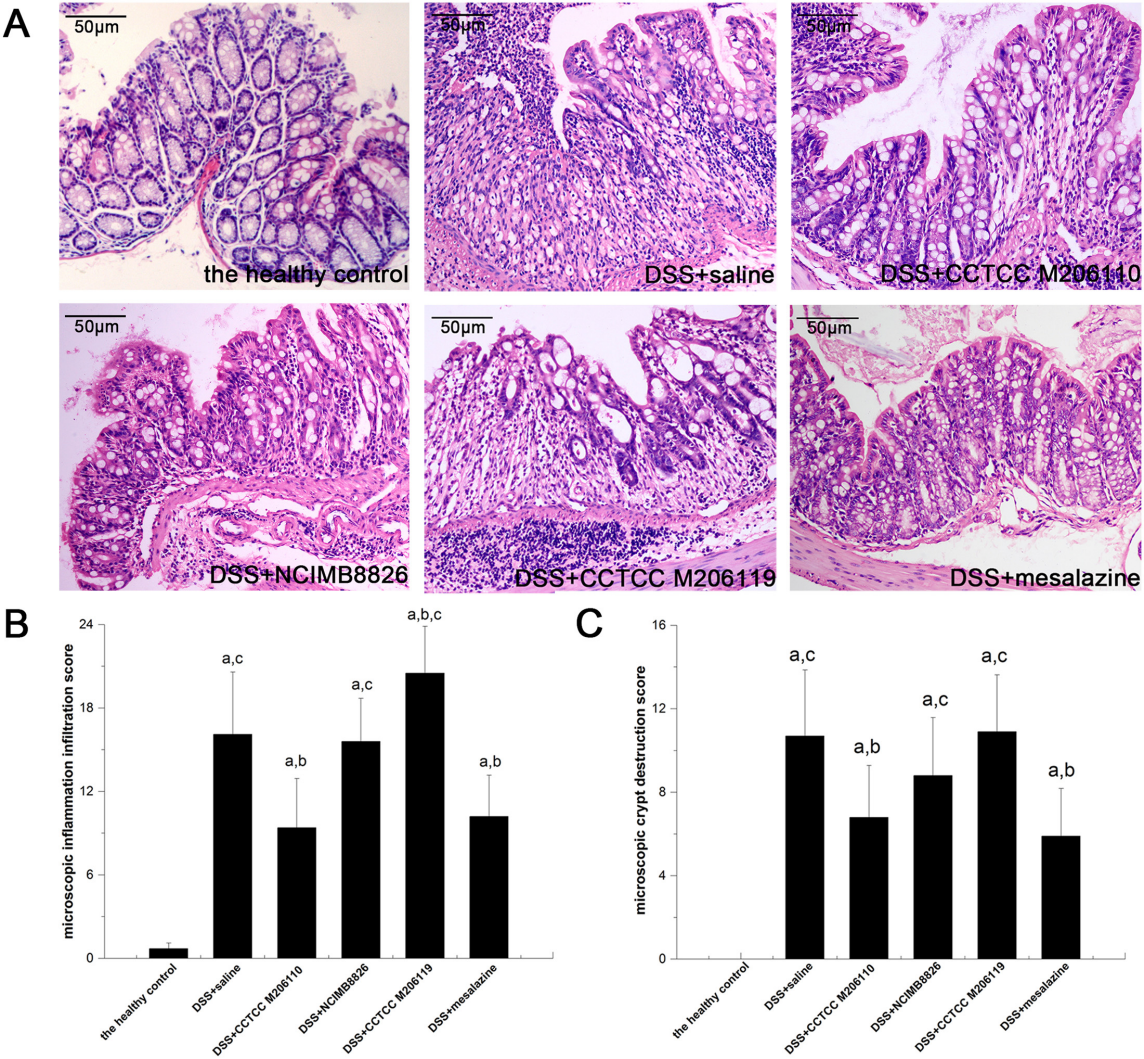
Effects of *Lactobacillus* strain treatments on colitis-induced changes in intestinal histopathologic features. (A) Histologic analysis of representative colons from the mice (original magnification, hematoxylin and eosin staining, 200×). (B) Microscopic inflammatory infiltration score; (C) Microscopic crypt destruction score. ^a^*P* < 0.05 vs. the healthy control group; ^b^*P* < 0.05 vs. the negative (saline) control group; ^c^*P* < 0.05 vs. the positive (mesalazine) control group.

The colon sections of the healthy control group showed intact mucosae with glands secreting abundant mucin. The colon sections of DSS-treated animals showed loss of the epithelial layer, goblet cell depletion, neutrophil infiltration, and distortion/destruction of the crypt architecture compared with the healthy controls; however, when the mice were co-treated with *L*. *fermentum*, the level of inflammatory infiltration was significantly lower compared with the negative control (9.40 ± 3.52 vs. 16.1 ± 4.48, *P* < 0.05), as was the extent of crypt structure destruction (6.80 ± 2.49 vs. 10.70 ± 3.16, *P* < 0.05). The results indicated no significant difference in the severity of inflammatory infiltration or crypt destruction between the *L*. *plantarum* group and the negative group (*P* > 0.05). Notably, more severe inflammatory infiltration was observed in the *L*. *crispatus* group compared with the negative controls (20.50 ± 3.37 vs. 16.1 ± 4.48, *P* < 0.05), with no significant difference in the severity of crypt destruction (Fig 3).

## Discussion

Therapies aimed at modifying and manipulating the gut flora have been implicated in the pathogenesis of UC through the use of probiotics and have received increased attention [19].

The application of *Lactobacillus* to UC is based on the evidence indicating the reduction of the fecal *Lactobacillus* count in UC patients [20–22]. In the current study, we demonstrated that the change in *Lactobacillus* in UC patients was more complicated at the species level. Not all *Lactobacillus* species were decreased in UC patients. The results of quantitative analysis of the 11 *Lactobacillus* species indicated that some *Lactobacillus* species increased significantly in UC patients compared with healthy controls, such as *L*. *crispatus*. Furthermore, the therapeutic effects of *Lactobacillus* strains were correlated with changes in the concentrations in the distal colon[23,24]. *Lactobacillus* strains of different species could exert completely opposite effects in UC.

Based on our study, *L*. *crispatus* numbers increased significantly in UC patients compared with healthy controls. The animal experiments further revealed that *L*. *crispatus* CCTCC M206119 aggravated weight loss and colonic histologic damage in the mouse colitis model. Previous studies on the effects of *L*. *crispatus* in induced colitis have reported conflicting results. Castagliuolo et al. [25] have demonstrated that *L*. *crispatus* M247 reduced the severity of DSS colitis in a dose-dependent fashion, while Ulinski and Aoun [26] revealed that the *L*. *crispatus* CCTCC M206119 strain is involved in the exacerbation of intestinal inflammation in DSS-colitis mice. Thus, we should be more cautious in administering *Lactobacillus* to UC patients in clinical practice.

The results obtained in the present study reveal that *L*. *fermentum* CCTCC M206110 appeared to be effective at attenuating DSS-induced colitis in BALB/c mice, given that the important clinical parameters, such as mortality, weight loss, DAI scores, and colonic histologic damage were improved in mice receiving *L*. *fermentum* compared with the controls. A previous study demonstrated that mice with colitis treated with *L*. *fermentum* had an improved survival rate, DAI score, and colonic mucosa histologic scoring [27]. The study further indicated that *L*. *fermentum* attenuated trinitrobenzenesulfonic acid-induced colitis, which was associated with an increase in intestinal superoxide dismutase activity and a reduction in oxidative stress, nuclear factor κB (NF-κB) activity, and cytokine production [28,29]. Our study tested the effect of *L*. *fermentum* CCTCC M206110 in DSS-induced colitis because of a previously noted reduction in *L*. *fermentum* species in UC patient feces. The present study indicates that *L*. *fermentum* exhibited beneficial anti-oxidative and anti-inflammatory properties in the mouse colitis model. Thus, a *L*. *fermentum* probiotic could be recommended as potential adjuvant therapy in combination with olsalazine to achieve a more efficacious treatment for UC. Nevertheless, because the associated clinical data are rather limited and far from convincing, the concomitant use of *L*. *fermentum* warrants well-designed, large, randomized, placebo-controlled trials to investigate the unresolved issues related to efficacy, dose, duration of use, and single or multistrain formulation.

In the current study, we failed to observe an obvious attenuation effect of *L*. *plantarum* NCIMB8826 in DSS-induced colitis in BALB/c mice. The results showed that *L*. *plantarum* NCIMB8826 improved weight loss and colon length, but with no significant influence on the DAI or colonic histologic damage in the mouse colitis model. During the last decade, the therapeutic effect of *L*. *plantarum* strains on ameliorating experimental colitis in mouse models has been reported [30–32]. Additionally, the anti-inflammatory and immunomodulatory activities of *L*. *plantarum* were evaluated, including reducing the production of pro-inflammatory cytokines (tumor necrosis factor (TNF)-α, interleukin (IL)-1, and IL-6) and inhibiting the Toll-like receptor (TLR)4-linked NF-κB and mitogen-activated protein kinase (MAPK) signaling pathways [31]. Furthermore, *L*. *plantarum* exhibited antioxidant properties by significantly decreasing lipid peroxidation (TBARS) and nitric oxide production and increasing the glutathione concentration [30,33]. It is arbitrary and inexact to draw the conclusion that *L*. *plantarum* is ineffective in DSS-induced colitis based on the present study, given the strong possibility of a false-negative result due to an insufficient sample size. Thus, the potential probiotic effect of *L*. *plantarum* on UC must be further assessed.

Hence, it is of great importance to establish the mechanism(s) underlying the beneficial interaction between the colonic epithelium, intestinal immunity, and the microbiota for the extensive but evidence-based application of probiotics in UC. Based on the previous studies, the gut microbiota plays a role in shaping the mucosal immune system [34]. *Clostridia* and *Bacteroides* have been proven to attenuate intestinal inflammation in mice through the induction of CD4^+^FOXP3^+^ regulatory T lymphocytes [35–37]. Other members of the microbiota can attenuate mucosal inflammation by antagonizing the activation of NF-κB or modifying the cytokine status [37].

In conclusion, the change in *Lactobacillus* in UC patients was more complicated at the species level. Not all *Lactobacillus* species are beneficial for UC patients. Administration of *L*. *crispatus* CCTCC M206119 supplement aggravated DSS-induced colitis, while *L*. *fermentum* CCTCC M206110 proved to be effective at attenuating DSS-induced colitis. *L*. *plantarum* NCIMB8826 showed no obvious attenuation effect in DSS-induced colitis. Thus, the potential probiotic effect of *L*. *plantarum* species on UC has yet to be assessed. Future studies should aim to determine the mechanisms underlying the interactions between *Lactobacillus*, the colonic epithelium and intestinal immunity, which may be essential for the extensive but evidence-based application of probiotics in UC.

## Supporting Information

S1 Table Colon length, weight loss, and disease activity index used in the study. (DOCX)
