# Supplementary material for: Different Effects of Three Selected Lactobacillus Strains in Dextran Sulfate Sodium-Induced Colitis in BALB/c Mice
Source: PLoS One. 2016 Feb 3;11(2):e0148241. doi: 10.1371/journal.pone.0148241 (PMC4739695; doi:10.1371/journal.pone.0148241)
Supplement: S1 Table — (DOCX) [file pone.0148241.s001.docx]

**S1 Table. Data of colon length,weight loss,disease active index used in the study**

| Group | n | Colon length (cm) | Weight loss (g) | DAI score |
| --- | --- | --- | --- | --- |
| The healthy conrol | 10 | 11.0±1.10 | 0.31±0.60 | 0.13±0.09 |
| saline | 8 | 6.71±1.47 | − 1.84±0.72 | 3.57±0.32 |
| CCTCC M206110 | 10 | 9.22±1.69 | − 0.82±0.39 | 3.30±0.33 |
| NCIMB8826 | 8 | 7.95±1.19 | − 1.19±0.55 | 3.77±0.35 |
| CCTCC M206119 | 5 | 5.25±1.19 | − 2.26±0.51 | 2.70±0.48 |
| mesalazine | 10 | 9.18±1.39 | − 0.71±0.28 | 2.77±0.63 |

Values are mean ± SD
